# Modulation of Peak Alpha Frequency Oscillations During Working Memory Is Greater in Females Than Males

**DOI:** 10.3389/fnhum.2021.626406

**Published:** 2021-04-23

**Authors:** Tara R. Ghazi, Kara J. Blacker, Thomas T. Hinault, Susan M. Courtney

**Affiliations:** ^1^Department of Psychological & Brain Sciences, Johns Hopkins University, Baltimore, MD, United States; ^2^Naval Medical Research Unit Dayton, Dayton, OH, United States; ^3^INSERM-EPHE-UNICAEN, U1077, Neuropsychologie et Imagerie de la Mémoire Humaine, Caen, France; ^4^Department of Neuroscience, Johns Hopkins University, Baltimore, MD, United States

**Keywords:** alpha oscillations, EEG – electroencephalogram, peak alpha frequency, sex differences, working memory

## Abstract

Peak alpha frequency is known to vary not just between individuals, but also within an individual over time. While variance in this metric between individuals has been tied to working memory performance, less understood are how short timescale modulations of peak alpha frequency during task performance may facilitate behavior. This gap in understanding may be bridged by consideration of a key difference between individuals: sex. Inconsistent findings in the literature regarding the relationship between peak alpha frequency and cognitive performance, as well as known sex-related-differences in peak alpha frequency and its modulation motivated our hypothesis that cognitive and neural processes underlying working memory—modulation of peak alpha frequency in particular—may differ based upon sex. Targeting sex as a predictive factor, we analyzed the EEG data of participants recorded while they performed four versions of a visual spatial working memory task. A significant difference between groups was present: females modulated peak alpha frequency more than males. Task performance did not differ by sex, yet a relationship between accuracy and peak alpha frequency was present in males, but not in females. These findings highlight the importance of considering sex as a factor in the study of oscillatory activity, particularly to further understanding of the neural mechanisms that underlie working memory.

## Introduction

Oscillatory activity within the alpha range of frequencies, canonically defined as 8–14 Hz, has been studied for its role in facilitating working memory (Klimesch, 1999; Roux and Uhlhaas, 2014). One critical characteristic of alpha activity – its peak frequency – has been tied to differences in working memory performance and is well known to differ across individuals based upon genetics, age, and the type of cognitive activity in which an individual is engaged (Posthuma et al., 2001; Mierau et al., 2017; Knyazeva et al., 2018). Despite these known differences, individuals are often treated as members of a single homogenous study group; their separate data points represented solely by group-level statistics. Here, we show that by accounting for an individual’s binary sex (female or male) differences in task-related modulation of peak alpha frequency, and its relationship to working memory performance become apparent.

### Alpha Oscillations and Working Memory

Oscillatory activity is thought to be one of the mechanisms by which functional organization and structured communication in the brain are achieved (Buzsáki et al., 2013; Voytek and Knight, 2015; Basar and Düzgün, 2016).

In working memory, the power of alpha oscillations has been related to performance (Klimesch, 1999), working memory maintenance (Herrmann et al., 2004), and working memory capacity (Tuladhar et al., 2007). Working memory by definition reflects rapid and short-lived processes that are subject to interference (Courtney et al., 2007). An ascribed functional role of alpha oscillations arising during working memory maintenance, is to facilitate preservation of maintained information. This facilitation is achieved through minimization of potential interference by dampening incoming external sensory stimuli when previously encoded information is being maintained (Jensen and Mazaheri, 2010; Ikkai et al., 2014; de Vries et al., 2020). The role of these oscillations may not simply be reactive, but proactive, as their phase and power have been found to shift in advance of anticipated distractors during a working memory task (Bonnefond and Jensen, 2012). Increases in posterior alpha power in particular, are hypothesized to reflect neural processes that protect information being held in working memory from interference by current sensory inputs (Klimesch, 2012; Ikkai et al., 2014; Roux and Uhlhaas, 2014). Causal manipulations using transcranial alternating current stimulation and transcranial magnetic stimulation at alpha frequencies supports this hypothesis (Borghini et al., 2018; Riddle et al., 2020).

### Peak Alpha Frequency

The alpha oscillatory activity of a neurotypical young to middle-age adult, measured when they are awake in a resting state, will on average exhibit a peak of power near the frequency of 10 Hz (Posthuma et al., 2001; Clark et al., 2004; Haegens et al., 2014). This apex of the power frequency distribution, known as the Peak Alpha Frequency (PAF), is known to be somewhat variable across individuals, however (Doppelmayr et al., 1998). Developmental studies have found that PAF increases throughout childhood and adolescence and only stabilizes at a frequency average near 10 Hz in late adolescence to early adulthood (Smith, 1941; Chiang et al., 2011). With aging, PAF will typically drift lower once again, with the average PAF of those near 70 years old being closer to 8 Hz (Dustman et al., 1999; Scally et al., 2018). The typical developmental trajectory of increasing working memory performance and capacity, followed by subsequent advanced-age declines (e.g., Park et al., 2002; Hamilton et al., 2003; Reuter-Lorenz and Park, 2010; Hinault et al., 2020), occur in seeming parallel to these described increases and decreases in PAF.

Peak alpha frequency can also shift on a timescale of moments, rather than that of a lifespan. During engagement in a cognitive task, PAF may shift to higher or lower frequencies, and do so differently across different brain regions. For example, Haegens et al. (2014) found that average posterior PAF increased when participants were performing an *n-back* working memory task compared to when they were at rest. They also found PAF increased with greater task difficulty. Sauseng et al. (2005) contrasted occipital and prefrontal PAF during sequential split-halves of a working memory task maintenance period. During working memory maintenance, PAF measured over posterior regions was consistently higher than PAF measured pre-frontally. When the task demanded information also be manipulated, however, for the first half of the analyzed time period prefrontal and posterior PAF were aligned. Their findings support different functional roles of alpha oscillations dependent upon the region from which they arise and the networks with which they are associated. Faster posterior alpha oscillations seemingly support working memory maintenance, while prefrontal support working memory control.

### Influences of Sex on PAF

Sex-linked differences in PAF have been reported starting in childhood and continuing on into advanced age. A developmental study of children, for example, found that males reached a higher and more stable average PAF than females by the age of 11 (Matthis et al., 1980). A more recent study found this difference continued up until the age of 16 (Chiang et al., 2011). A large study that utilized a clinical database with EEG recordings from patients ranging in age from infancy to the late 80s, found that from 20 years of age and continuing up to 85, females had higher alpha frequencies than males (Aurlien et al., 2004).

Studies from the 1970s onwards have demonstrated predictable sex-linked variance in PAF. Wuttke et al. (1975), for example, measured resting state alpha frequencies in two groups of women: one naturally cycling, and one taking hormone-based oral contraceptives. In the naturally cycling group, alpha frequencies were found to increase and decrease with the cyclical fluctuation of sex- hormones that occur across menstrual phases. In the group taking oral contraceptives, which prevent natural sex-hormone level fluctuations, alpha frequencies remained relatively constant over time (Wuttke et al., 1975). Becker et al. (1982) also examined the connection between sex-hormones and resting-state alpha changes across the menstrual cycle in females, but also included cognitive task performance as a variable of interest. Their psychological battery included tests of short-term and working memory. Replicating the findings of Wuttke et al. (1975), they found that mean alpha frequency increased and decreased cyclically in conjunction with fluctuating hormone levels, yet they found no difference in cognitive performance tied to these fluctuations (Becker et al., 1982).

### PAF and Cognitive Performance

The lack of relationship between sex-linked PAF fluctuations and performance described above might be surprising considering the positive associations between PAF and behavior previously reported. Higher resting state anterior PAF has been linked to higher *Reverse Digit Span* performance, for example, independent of age in a large gender-balanced study (Clark et al., 2004). In another study comprised of 12 males and 4 females, the researchers employed a split-half analysis of working memory performers and found that the average PAF of the good performer group was 1.25 Hz greater than that of the poor performer group (Klimesch et al., 1993). Additional evidence for positive correlations between PAF and speed of information processing, and PAF and memory performance have also been reported (Klimesch et al., 1990; Bazanova and Vernon, 2014). Moreover, shifting PAF higher or lower using sensory entrainment has been shown to causally influence cognitive performance (Ronconi et al., 2018).

In other cases, however, the relationship between PAF and performance is less clear. Angelakis et al. (2004), for example, reported no PAF-performance relationships in a small gender-balanced experiment. In a follow-up experiment where PAF and task performance were measured on two separate days in 13 females and 6 males, they found correlations of posterior PAF with performance on 1 day, but not the other (Angelakis et al., 2004). A possible contributor to the discrepant findings between all of these studies considered may be the neglect of a key factor – biological sex. Indeed, there is a gap in the literature regarding potential sex differences in PAF, particularly potential differences while under cognitive load.

### Study Question

We hypothesized that during working memory, females and males may differentially modulate oscillatory neural activity to support task performance. As discussed above, females of reproductive age exhibit predictable sex-specific variation in PAF, yet this variation does not necessarily impact their task performance. In some studies that included both male and female participants, relationships between PAF and performance have been found. It is possible, therefore, that cognitive and neural processes related to alpha oscillatory activity underlying working memory, are utilized differently in females versus males, particularly as they contribute to task performance. These differences may be tied to sex differences in PAF modulation, or differences in the relationship between individual variability in PAF and working memory performance.

### Materials and Methods

To test our hypothesis, we re-analyzed data from a study that employed a task in which both female and male participants maintained different types of spatial information in working memory – either precise locations of individual stimuli or spatial locations of the stimuli relative to each other– while undergoing EEG (Blacker et al., 2017). Brain activity during working memory for these different types of information has been found to differ, both neuroanatomically as measured with fMRI (Blacker and Courtney, 2016) and electrophysiologically as measured with EEG (Blacker et al., 2016). We hypothesized, therefore, that there might be sex differences in the neural activity underlying working memory for both precise and relative locations, only precise, only relative, or neither.

Analysis of neural activity recorded by EEG during key working memory periods – particularly maintenance and baseline– enabled us to compare PAF across both time periods and task conditions. It also enabled examination of a possible differential relationship between PAF and behavioral performance for females versus males. A recent meta-analysis reported a small female advantage in visuospatial working memory (Grissom and Reyes, 2019) whereas previous meta-analyses have found a small male advantage in certain types of spatial tasks (Hyde, 2014). The paradigms employed in those studies, however, differ from the paradigm used here, and behavioral similarities between females and males are the more typical finding (Hyde, 2005). Hence, we predicted there would be no difference between females and males in behavioral performance. We did, however, predict differences between these groups in PAF, given the prior evidence outlined above. Further, we predicted that within-group differences in PAF or task-related modulation of PAF might be tied to behavioral performance, but that this activity-behavior relationship might be different in females versus males.

### Participants

Study participants were young adults recruited from both the college student population and the local community. Participants ranged in age from 18 to 31 years. Binary grouping of participant sex as female or male was based upon self-report.

Any participants with below chance behavioral performance in the primary task, or incomplete EEG data, were excluded. After these criteria were applied, data from a total of 110 participants (33 males and 77 females) remained and were analyzed. Groups were not statistically different in age [*p* > 0.3; mean (SD): males 21.8(3.4), females 21.1(3.2) years].

### Working Memory Task

**FIGURE 1:**
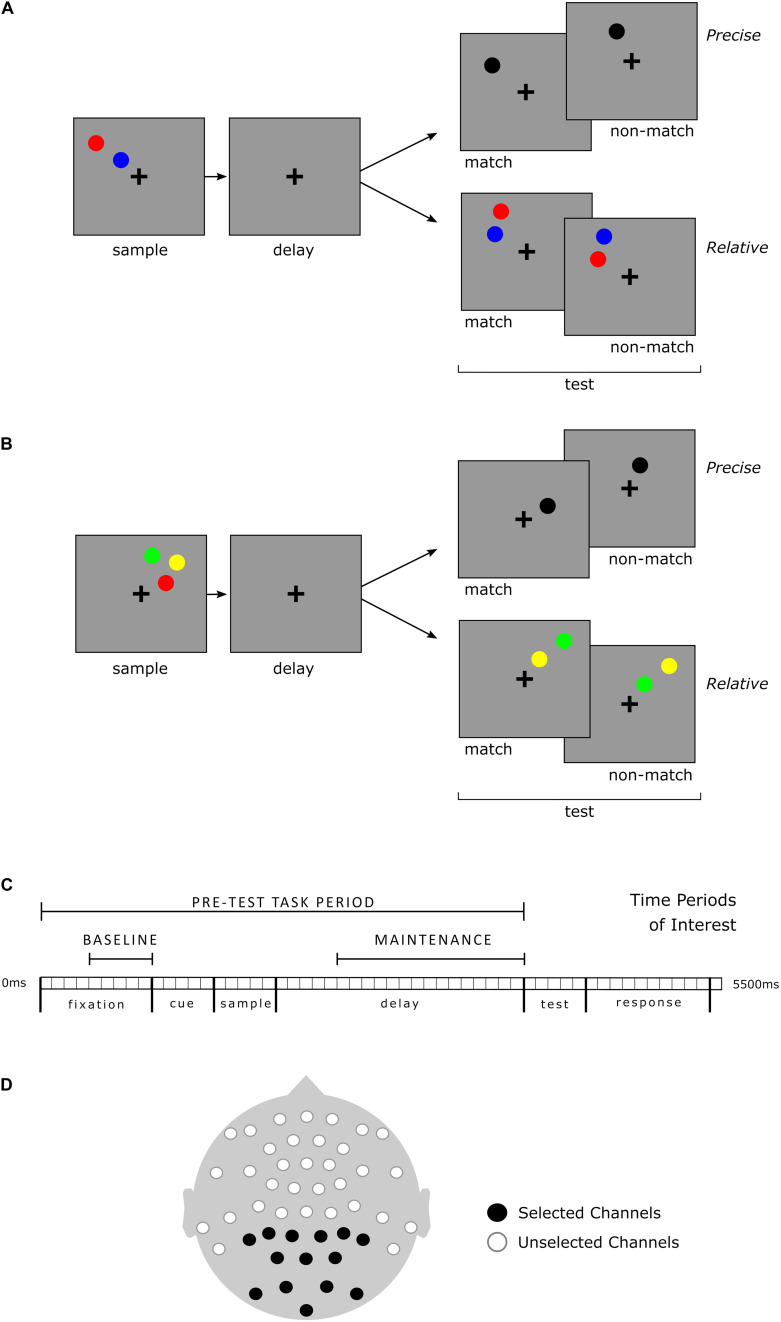
Working memory task and timeline, selected time periods and channels for analysis of EEG. The four distinct Task-types included two or three sample stimuli within Precise and Relative conditions. All Task-types required a match/non-match decision based upon the specific instructions for the Task-type. Visualization is organized for clarity but note that sample load is not an independent factor from condition; instructions differed for each of the four Task-types. **(A)** 2-Sample Task-type examples. In the Precise spatial Task-type, the test stimulus (black) matched if positioned upon an imaginary line segment (held in working memory) connecting the centers of the two sample stimuli. In the Relative spatial Task-type, the vertical spatial relationship of the colored test stimuli was relevant, but not their specific positions. In the example shown, the red sample stimulus is positioned higher than the blue sample stimulus. At test, a red stimulus above a blue stimulus would be a match, regardless of their particular vertical or horizontal positions. **(B)** 3-Sample Task-type examples. With three sample stimuli, in the Precise Task-type, the test stimulus was a match if it occupied the exact spatial position of any one of the sample stimuli. In the Relative task-type, test stimuli could be any two of the three sample stimuli colors, but were always in a different spatial location (though within the same visual quadrant); only the vertical spatial relationship between the corresponding color stimuli was relevant. **(C)** Timeline for each Task-type and time periods of interest for EEG analysis. Baseline: 500 ms prior to cue and visual sample stimuli presentation (the cue indicated whether a precise or relative judgment would be required). Maintenance: 1,500 ms during the working memory delay prior to presentation of test stimuli. **(D)** EEG channel montage. All recorded channels are depicted. The 14 channels indicated in black were selected *a priori* for PAF analyses. All 14 selected channels were included in the analysis for each time period of interest.

A visuospatial working memory task (Figure 1) was performed while continuous EEG was recorded. Conditions (Task-types) were distinguished based upon the number of sample stimuli presented and what aspects of those stimuli were relevant. Instructions differed for each of the four Task-types but are described here first based upon their similarities to aid comprehension. In Precise location trials the specific locations of the sample stimuli were relevant, whereas in Relative trials, the locations of the sample stimuli relative to each other were relevant. Either two or three colored circle stimuli were presented for 500 ms during the sample period. Instructions for each of the four Task-types are as follows: in 2-Sample Precise trials, participants were instructed to imagine a single line connecting the two sample stimuli, and then, after a 2,000 ms delay, determine if the test stimulus, a single black circle, was located upon that imagined line. In 3-Sample Precise trials, participants were to determine if the test stimulus occupied the same location as any one of the sample stimuli. In 2-Sample Relative trials, participants were to maintain the vertical relationship between the sample stimuli and determine if the test stimuli, also two, colored circles, held the same relationship. In 3-Sample Relative trials, the vertical spatial relationship between any two of the three sample stimuli might be relevant at test. All stimuli were displayed in the same single quadrant of the screen during a trial. Participants were instructed to maintain fixation on a central cross for the duration of each trial.

Participants were able to indicate a match or non-match response by keypress as soon as the test stimuli were displayed, and for up to an additional 1,000 ms after. Feedback for each trial was presented via a 100 ms color change in the on-screen fixation cross and was immediately followed by the start of the next trial.

Prior to the EEG recording session, participants completed 88 practice trials, 22 of each Task-type. During EEG recording, participants completed 8 blocks of 64 trials for a total of 512 trials. Task-type trial order was pseudorandomized within each block. For further, detailed task methods, see *Spatial Locations and Relations Task* (Blacker et al., 2017; Hinault et al., 2019).

### EEG Collection, and Pre-processing

The EEG recording was performed at a sampling rate of 512 Hz with a 138 Hz cutoff filter. A 47-electrode equidistant montage was utilized and re-referenced to the average of all channels offline (for further details of EEG acquisition, see Blacker et al., 2017). The Fieldtrip software package (Oostenveld et al., 2011) was utilized for raw EEG data cleaning, processing, and analysis. Per the Fieldtrip pipeline, raw data were segmented, cleaned, and sorted by Task-type prior to spectral decomposition for analysis of induced power in the time-frequency domain. Detailed steps of this procedure follow.

Prior to segmentation, the continuous raw EEG data was high-pass filtered at 0.5 Hz, de-meaned, and low-pass filtered at 30 Hz. Then, data from each of the 47 channels were divided into temporally overlapping 7.5 s segments that included the 5.5 s trial period and the 2 s preceding trial onset. This is described as overlapping due to the final 2 s of one segment being the same as the starting 2 s of the subsequent segment. This standard approach of buffering via extended segmentation (Cohen, 2014) ensured preservation of signal during the Baseline time period (ref Figure 1). Independent Components Analysis with 40 primary components (Oostenveld et al., 2011) was utilized to identify, and target for removal, artifacts resulting from blinks, eye movements or excessive facial muscle activity (e.g., jaw clenching). Following this algorithm-based cleaning, visual inspection of the EOG channels placed above the left and right eyes was performed to identify ocular artifacts. Trials containing voltage shifts greater than 18.75 μV in the EOG channels were removed from the data. Finally, any remaining trials with large (e.g., an order of magnitude greater) voltage spikes not filtered out by independent component analysis were removed.

Clean, segmented data were sorted based upon Task-type. Only correct trials were selected for analysis. In preparation for subsequent frequency-based analyses, spectral decomposition was performed. Cleaned, sorted, correct trial data underwent spectral analysis at a frequency resolution of 0.5 Hz from 2.0 to 20.0 Hz via complex wavelet convolution with a 2.0 s sliding window, zero-padded with a Hann/hanning multi-taper time frequency transformation based upon multiplication in the frequency domain. The Hann taper is well-suited for broad-band low frequency estimation and does not introduce edge artifacts (Cohen, 2014). This approach allowed for measurement of power at precise and specific frequency increments, with sufficient frequency resolution for direct comparative analyses between the defined time periods of interest. Spectral analysis resulted in a 3-dimensional data matrix of power organized by time, frequency, and channel. This matrix was utilized for the analyses described below.

### Peak Alpha Frequency

The *Center of Gravity* method was used for calculating Peak Alpha Frequency (PAF), (Klimesch, 1999; Goljahani et al., 2012). In this method upper and lower frequency bounds and a time period are used as constraints within which to establish the specific frequency at which the power of alpha oscillations is maximal. This method was selected for its potential to increase the signal-to-noise ratio when calculating PAF during a cognitive task (Klimesch et al., 1993; Klimesch, 1999). The canonical alpha range of 8–14 Hz was used to define the frequency boundaries within which PAF was calculated and three time periods of interest were selected.

As discussed in the introduction, alpha power arising from posterior brain regions is understood to facilitate working memory maintenance. Posterior electrode channels are a common target for measurement of PAF (e.g., Haegens et al., 2014). Additionally, this study incorporated a visual working memory paradigm in which there have been prior findings related to alpha oscillatory activity over posterior regions (Blacker et al., 2016). For the purposes of this study, therefore, of the 47 channels recorded, 14 posterior channels were selected *a priori* for PAF calculation and used in entirety for calculation of PAF during each time period of interest. Channels and time periods are shown in Figure 1. *A priori* channel selection allowed for direct comparison of participants based upon female or male grouping (see recommendations in Rippon et al., 2014) whereas a data driven approach might suffer confounds resulting from unforeseen sex-linked group differences in electrode distribution or gross brain anatomy.

Channels and time periods for analysis were selected with the aim of minimizing inclusion of (if not excluding) signal not primarily related to alpha and working memory processes of particular interest in this study. Mu band activity, for example, overlaps with the alpha frequency range but originates in relation to motor response and is typically measured over motor and pre-motor regions (e.g., Hadley, 1941). Mastoid channels and the central topmost channel, which due to their position may be detecting activity related to motor response rather than working memory maintenance, were thus not included in the selection of posterior channels. Additionally, the time periods of interest (ref Figure 1) did not include the Test or Response periods during which stimuli differed across Task-types and participants were preparing for, or making, a button press response.

Three separate measures of PAF within the range of 8.0–14.0 Hz at a frequency resolution of 0.5 Hz for each participant were made: during Baseline, Maintenance, and across the Pre-Test Task period (which included the Baseline and Maintenance periods as shown in Figure 1). Two discrete time periods of interest during the task, labeled Baseline and Maintenance were targeted for comparative PAF analyses. Baseline was defined as the last 500 ms of fixation, prior to the presentation of the cue and sample stimuli. The length of this time window allowed for measurement of approximately four oscillatory cycles at the lowest frequency of interest here (8 Hz) – a number of cycles within the typically recommended range (Cohen, 2014). This Baseline period covers a pre-stimulus range of time similar to the baseline periods of other studies (e.g., Blacker et al., 2016; Hinault et al., 2019). Maintenance was defined as the last 1,500 ms of the delay, prior to the presentation of test stimuli. As the name of the time period suggests, this period allows for analysis of PAF as it relates to the maintaining of information in working memory. The naming and definitions of these time periods of interest is in line with previous working memory research that utilizes EEG (e.g., Manza et al., 2014; Blacker et al., 2016; Bae and Luck, 2018; Hinault et al., 2019; and see Roux and Uhlhaas, 2014).

As stated earlier, spectral analysis results in a 3-dimensional matrix. Within that matrix, power is organized by the channel measured, the frequency at which oscillation is occurring, and the time of measurement. Following the *Center of Gravity* method (Klimesch, 1999), to attenuate noise from any single one of the 14 posterior channels selected, a weighted averaging of power across channels was performed prior to PAF calculation. To compute weights for the weighted averaging process, power across the 14 channels of interest was first summed at a single time point. The proportion of a single channel’s contribution to that sum was deemed its weight at that time point. At each time point, and for each 0.5 Hz frequency increment within the range of 8–14 Hz, the vector of power for each channel and the vector of weights for each channel were multiplied together. The average of this product was then taken. These steps were performed for all frequencies and time periods of interest. The frequency at which the largest weighted average of power existed within the time period of interest was denoted the Peak Alpha Frequency.

### Statistical Analysis

Dependent variables relevant to our predictions included Accuracy (proportion of correct responses), Response Time, PAF, and PAF Modulation. Both parametric and non-parametric analytical approaches were employed with these measures defined as outcome variables. To test our predictions regarding task performance, PAF, and PAF Modulation, group level distributions were statistically compared using a robust and non-parametric approach: the Kolmogorov–Smirnov test (Rousselet et al., 2017). Modulation of PAF between the Baseline and Maintenance time periods was calculated as [Maintenance PAF – Baseline PAF] in units of Hz.

In addition to null hypothesis testing for a difference in distributions, shift functions (Doksum, 1974; Doksum and Sievers, 1976) were computed. This method quantifies how two distributions differ by comparing corresponding quantiles of each distribution and delivering a function showing how one of the distributions would need to change, or “shift,” to match the other. All shifts between quantiles are statistically tested as a group with correction for multiple comparisons, providing a robust measure of where and how distributions differ (Doksum and Sievers, 1976; Rousselet et al., 2017). All shift functions calculated herein computed differences between the Male and Female distributions as a function of the Female distributions. Further description and illustration of this method are provided in detail elsewhere (Rousselet et al., 2017).

Parametric group level comparisons of Accuracy, Response Time, and PAF Modulation by Task Type were performed with separate 2 (Sex) × 4 (Task-type) ANOVA tests. This 2 × 4 analysis was more appropriate than a 2 × 2 × 2 ANOVA design because task instructions differed for each Task-type dependent upon load and thus load was not an independent factor. In addition to this statistical rationale, our previous findings pointed toward a qualitative difference in the neural effects of load for Precise versus Relative Task-types in this paradigm (Blacker and Courtney, 2016). For completeness, however, we also analyzed the data using a 2 × 2 × 2 ANOVA. The results of the two analyses were essentially identical. In the results below, we present the 2 × 4 ANOVA results. In cases where violations of sphericity were present, correction was applied and reported. Tukey’s Honestly Significant Difference (HSD) method at an alpha level of 0.05 was used in *post hoc* analyses. For models of task performance and PAF Modulation, main effects of Sex, Task-type, and their interaction were fit.

To test our prediction of differential relationships between behavior and PAF, and behavior and PAF Modulation, between Females and Males, hierarchical multiple regression and correlational analyses were performed. Accuracy (proportion correct) and Response Time were the behavioral measures of interest and the outcome variables for all equation-based analyses. Baseline, Maintenance, and Pre-Test Task period PAF—encompassing both the Baseline and Maintenance time periods—was utilized in modeling. Separate two-step hierarchical regressions were performed for each group due to the prediction of different relationships by Sex in which PAF impacts behavior. In the first step, Task Performance (Accuracy and Response Time were each modeled separately) was predicted by Task-type. In the second step, PAF (Pre-Test Task PAF, Baseline PAF, and Maintenance PAF were modeled separately) was added into the model as an interacting factor. An ANOVA test was then used to determine if the first and second models differed significantly. In *post hoc* correlational analyses, PAF from each of the three time periods of interest was used. The False Discovery Rate (FDR) method was used for multiple comparisons corrections of *p*-values.

Statistics were calculated in R (R Core Team, 2018) using the *dplyr* (Wickham et al., 2020), the *car* (Fox et al., 2012), the *ez* (Lawrence, 2016), and the *lme4* (Bates et al., 2015) packages. Distribution analyses were performed using Robust Graphical Methods For Group Comparisons (the *rogme* package), per the methods described in Rousselet et al. (2017). Figures were produced in R with the *ggplot2* (Wickham, 2016) and the *rogme* packages.

## Results

Behavioral results are presented first. They are followed by results of analyses of PAF calculated from correct trials of all Task-types (Precise and Relative, 2- and 3-Sample stimuli), and then results of analyses with PAF calculated separately for each Task-type.

### Task Performance

Behavioral measures of central tendency are reported in Table 1. Two separate 2 × 4 (Sex × Task-type) ANOVAs were performed with Accuracy and Response Time as outcome variables. Main effects of Task-type were present for Accuracy: *F*(3,324) = 356.35, *p* < 0.0001, η^2^ = 0.546, and Response Time: *F*(3,324) = 182.68, *p* < 0.0001, η^2^ = 0.217. Accuracy was lower for the higher load (3-Sample) Task-types. Response time was greater for the Relative Task-types as has been previously observed (Ikkai et al., 2014). No main effects of Sex, nor interaction between Task-type and Sex were present. *Post hoc* analysis of Accuracy indicated significantly different groupings between 3-Sample Precise and 3-Sample Relative Task-types, and each in comparison to the 2-Sample Task-types together. No differences were present by Sex. *Post hoc* analysis of Response Time indicated significantly different groupings between 2-Sample Precise compared to 3-Sample Relative and 3-Sample Precise Task-types, with no differences by Sex.

**TABLE 1:** Measures of central tendency—medians, means, and standard deviations (SD)—in task performance by group: Females (F) and Males (M), shown for each Task-type.

		**Task-type**											
		**2-Sample Precise**			**2-Sample Relative**			**3-Sample Precise**			**3-Sample Relative**		
	**Sex**	***Median***	***Mean***	***SD***	***Median***	***Mean***	***SD***	***Median***	***Mean***	***SD***	***Median***	***Mean***	***SD***
Accuracy *(proportion correct)*	F	0.93	0.91	0.08	0.95	0.92	0.07	0.70	0.72	0.09	0.88	0.86	0.08
	M	0.95	0.92	0.06	0.96	0.93	0.06	0.69	0.70	0.08	0.88	0.86	0.09
Response time *(ms)*	F	663.7	665.7	122.9	713.5	715.5	115.8	747.2	750.6	120.5	851.3	834.8	110.4
	M	668.6	641.6	132.6	673.7	681.4	141.7	738.1	722.2	135.6	835.7	830.7	132.1

*Main effects of Task-type are present for Accuracy (*p* < 0.05) and Response Time (p < 0.05). No main effect of Sex or interaction between Task-type and Sex are present.*

**FIGURE 2:**
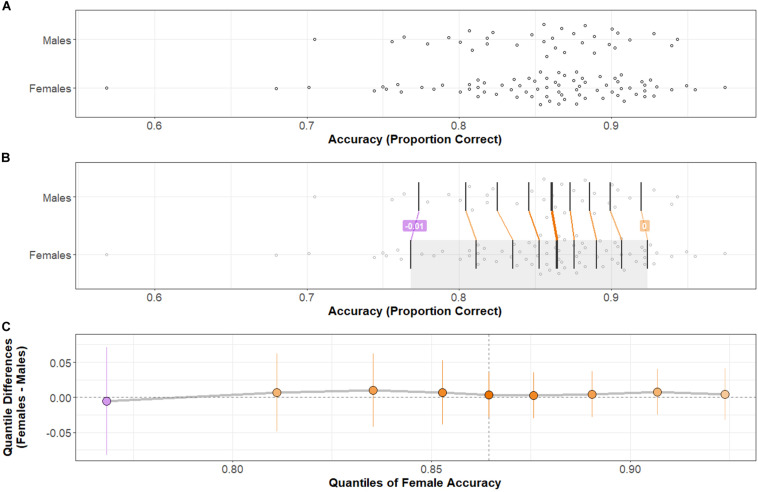
Accuracy distributions by group and comparison by shift function. **(A)** Stripchart of Accuracy distributions by group. **(B)** Stripchart shown in **(A)** with quantiles (vertical black bars) and differences between distribution quantiles characterized. When the difference between quantiles (Females–Males) is negative, the connecting line between corresponding quantiles is purple; when positive, orange. The heavier weight vertical black bar denotes the median (5th quantile) of each distribution. **(C)** Shift function between Male and Female distributions. Range of the *x*-axis corresponds to the shaded region of the Female distribution in **(B)**. *Y*-axis shows the difference between group distributions by quantile: as in **(B)**, purple indicates a negative difference; orange indicates a positive difference. Points indicate how much the Male distribution would need to shift at a particular quantile to match the corresponding quantile in the Female distribution. Vertical lines at each point represent a bootstrapped 95% confidence interval about the difference. Note that difference points all near zero and confidence intervals at every quantile difference point cross over zero: there is no significant difference in Accuracy across Task-types between Females and Males. Two-sample, two-sided Kolmogorov–Smirnov test: *D* = 0.09, *p* > 0.5.

**FIGURE 3:**
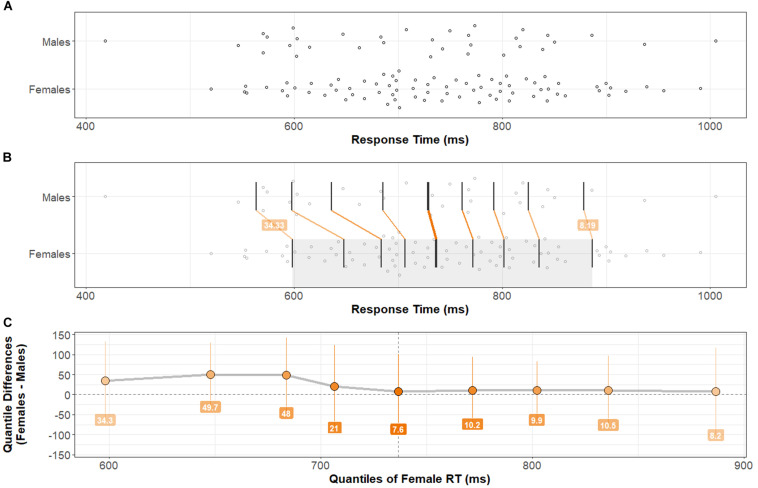
Response Time distributions by group and comparison by shift function. **(A)** Stripchart of Response Time distributions (mean Response Time across Trial-types) for Males and Females. **(B)** Stripchart shown in **(A)** with quantiles and differences between distribution quantiles characterized (see Figure 2 caption for detailed explanation). **(C)** Shift function between Male and Female distributions. Note that bootstrapped 95% confidence intervals at each quantile difference point cross zero. There is no significant difference in Response Time across Task-types between Females and Males. Two-sample, two-sided Kolmogorov–Smirnov test: *D* = 0.17, *p* > 0.4.

Group distributions and shift functions of Accuracy and Response Time across all correct trials are shown in Figures 2, 3, respectively. Two-sample, two-sided Kolmogorov–Smirnov tests indicated no difference between Female and Male Accuracy distributions: *D* = 0.09, *p* > 0.5; or Response Time distributions: *D* = 0.17, *p* > 0.4.

### PAF Within Each Time Period

The PAF was extracted for each participant during the time periods of interest. Measures of central tendency for PAF by Task-type and Sex are presented in Table 2. Separate 2 × 4 (Sex × Task-type) ANOVA tests were performed with PAF from the different time periods as outcome variables. Baseline PAF test results indicated no main effect of Sex: *F*(1,108) = 0.99, *p* > 0.3, Task-type: *F*(3,324) = 0.34, *p* > 0.7, nor an interaction between them: *F*(3,324) = 0.99, *p* > 0.3. With PAF during Maintenance as the outcome variable, test results indicated no main effect of Sex: *F*(1,108) = 0.24, *p* > 0.6, no significant main effect of Task-type: *F*(3,324) = 1.82, *p* > 0.1, nor an interaction between them: *F*(3,324) = 1.06, *p* > 0.3.

**TABLE 2:** Measures of central tendency—medians, means, and standard deviations (SD)—in peak alpha frequency (PAF) by group: Females (F) and Males (M).

		**Task-type**											
		**2-Sample Precise**			**2-Sample Relative**			**3-Sample Precise**			**3-Sample Relative**		
	**Sex**	***Median***	***Mean***	***SD***	***Median***	***Mean***	***SD***	***Median***	***Mean***	***SD***	***Median***	***Mean***	***SD***
Baseline	F	10.5	10.29	1.27	10.5	10.30	1.32	10.5	10.31	1.34	10.5	10.28	1.30
	M	10.5	10.67	1.34	10.5	10.42	1.14	10.5	10.53	1.39	10.5	10.56	1.12
Maintenance	F	10.5	10.47	1.22	10.5	10.63	1.18	10.5	10.63	1.18	10.5	10.74	1.18
	M	10.5	10.53	1.25	10.5	10.58	1.09	10.5	10.58	1.09	10.5	10.53	0.93
Modulation	F	0	0.18	0.89	0	0.33	1.13	0	0.38	1.40	0	0.46	1.12
	M	0	−0.14	0.69	0	0.15	0.61	0	−0.06	0.83	0	−0.03	0.72

*PAF during the Baseline and Maintenance time periods (as shown in Figure 1), and PAF Modulation are all presented in units of Hz.*

Two-sample, two-sided Kolmogorov–Smirnov tests were performed to test for differences between Female and Male distributions both averaged across, and within specific Task-types, during the Baseline and Maintenance time periods. Test results indicated no significant differences between group distributions at Baseline nor during Maintenance, *p* > 0.1 in all instances.

### Modulation of Peak Alpha Frequency

The PAF Modulation measures of central tendency by Task-type are shown in Table 2. As predicted, the results of a 2 × 4 ANOVA (Sex × Task-type) with Modulation as the outcome variable shows a main effect of Sex: *F*(1,108) = 5.31, *p* < 0.025, η^2^ = 0.03. No main effect of Task-type: *F*(3,324) = 1.56, *p* > 0.1, or interaction between Sex and Task-type: *F*(3,324) = 0.63, *p* > 0.5, was present. Females exhibited greater Modulation of PAF than Males, across Task-types.

Considering that Males Modulated PAF less than Females, it would be expected that PAF at Baseline would be more highly correlated with PAF during Maintenance among Males compared to Females. This was the case. For Males, Pearson’s *r* = 0.82, for Females: *r* = 0.58. The difference between Male and Female correlations is significant: *z* = 4.71, *p* < 0.0001.

**FIGURE 4:**
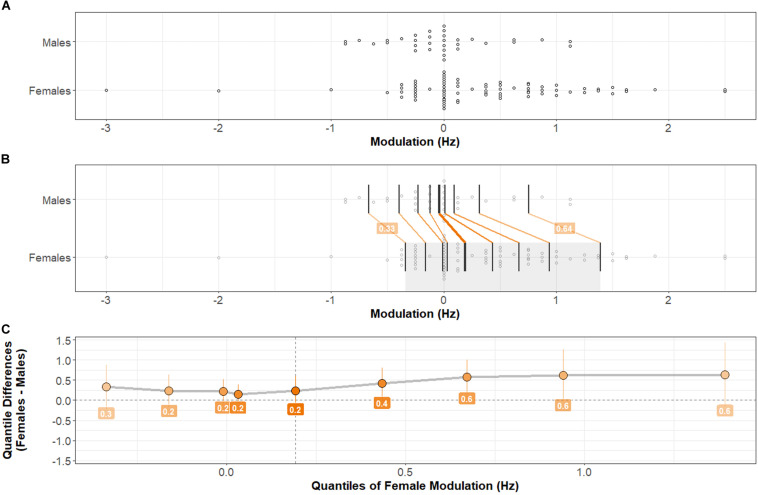
Distributions of PAF Modulation by Sex. Comparison between Male and Female distributions by shift function. **(A)** Stripchart of PAF Modulation distributions (mean Modulation across Task-types) for Males and Females. **(B)** Stripchart shown in **(A)** with quantiles and differences between distribution quantiles characterized (see Figure 2 caption for detailed explanation). **(C)** Shift function between Male and Female distributions. The Female distribution exhibits a positive shift at each quantile compared with the Male distribution. The difference is greatest in higher quantiles. Two-sample, two-sided Kolmogorov–Smirnov test: *D* = 0.29, *p* < 0.05. Females exhibit significantly more positive PAF Modulation than Males.

Distributions of Modulation of PAF (the difference, calculated within each individual, between PAF during the Maintenance versus Baseline time periods) are shown in Figure 4. A two-sample, two-sided Kolmogorov–Smirnov test indicates a statistically significant difference between the Female and Male distributions of Modulation: *D* = 0.29, *p* < 0.05.

### Peak Alpha Frequency and Behavior

To test our prediction of a relationship between PAF and task performance, regression analyses using behavioral measures (Accuracy and Response Time) as outcome measures predicted by PAF during the time periods of interest were performed. Separate correlational analyses were then performed to further examine the relationship between PAF and Accuracy for each Task-type.

Females exhibited greater Modulation of PAF than Males (above results) yet did not have an apparent behavioral advantage in performance across Task-types. It is possible that different relationships between PAF, Modulation of PAF, and task-performance exist for males versus females. Female and Male groups were modeled separately, based upon the prediction of different PAF-behavior relationships between them.

In all second-step hierarchical models with Response Time as the outcome variable, the addition of PAF as a predictor did not improve model fit above and beyond Task-type alone. This was the case for both Males and Females, and PAF from each time periods of interest.

For the Female group, the addition of PAF to Task-type as a predictor of Accuracy did not improve model fit over Task-type as a sole predictor. This was the case for PAF during all three time periods of interest (Baseline, Maintenance, and the Pre-Test Task period).

For the Male group, however, model fit improved significantly when PAF was incorporated as a predictor of Accuracy. In Males, Accuracy predicted by the interaction of Task-type and Baseline PAF was a better fit than Accuracy predicted by Task-type alone: *F*(2,124) = 3.30, *p* < 0.05. The second-step model incorporating Baseline PAF was a significant predictor of Accuracy: *F*(7,124) = 36.6, *p* < 0.0001, *R*^2^ = 0.66. The second-step model incorporating Maintenance PAF as an interacting predictor in Males, exhibited a trending difference over Task Type as the sole predictor: *F*(2,124) = 2.22, *p* < 0.08. This second-step model was also a significant predictor of Accuracy in Males: *F*(7,124) = 34.9, *p* < 0.0001, *R*^2^ = 0.64. As expected, then, given that the Pre-Test Task period incorporates both the Baseline and Maintenance period, the second-step model adding Pre-Test Task period PAF also proved a significantly better fit than the first-step model: *F*(2,124) = 4.07, *p* < 0.01. This second-step model was a significant predictor of Accuracy in Males: *F*(7,124) = 37.8, *p* < 0.0001, *R*^2^ = 0.66.

Further examining this relationship between Accuracy and PAF in Males, separate correlational analyses were performed. Correlations by group, separately for each Task-type and corrected for multiple comparisons, between Accuracy and PAF during the three time periods of interest are shown in Table 3. In Precise Task-types, there was no significant correlation between Accuracy and PAF for either Females or Males. A significant positive correlation between PAF and Accuracy among Males was present in the 3-Sample Relative Task-type (*r* = 0.49, *p* < 0.05) and trending in the same direction in the 2-Sample Relative Task-type (*r* = 0.42, *p* = 0.11) during the Pre-Test Task time period of interest. There was no correlation between these measures in Females (*r* = −0.01, *p* > 0.05). A significant difference was present between Male and Female group correlations in the 3-Sample Relative Pre-Test Task time period (*z* = 2.51, *p* = 0.01).

**TABLE 3:** Correlations between accuracy and peak alpha frequency (PAF) during the pre-test task period, baseline and maintenance time periods (as shown in Figure 1).

	**Pre-Test Task Period PAF**				**Baseline PAF**				**Maintenance PAF**			
	**Females**		**Males**		**Females**		**Males**		**Females**		**Males**	
**Task-type**	***r***	***p***	***r***	***p***	***r***	***p***	***r***	***p***	***r***	***p***	***r***	***p***
2-Sample Precise	0.05	–	0.02	–	0.08	–	−0.00	–	0.00	–	0.08	–
2-Sample Relative	0.13	–	0.40	0.14 (0.02)	0.09	–	0.37	0.14 (0.03)	0.13	–	0.30	0.23 (0.09)
3-Sample Precise	−0.09	–	0.08	–	0.10	–	0.05	–	−0.04	–	−0.00	–
3-Sample Relative	**−0.02**	–	**0.49**	0.03 (0.00)	0.03	–	0.44	0.04 (0.01)	−0.05	–	0.37	0.09 (0.04)

*Accuracy means by Trial-type are shown in Table 1. Pearson’s r correlation statistics are shown along with p-values indicating significance of difference from zero. All p column values of “–” indicate a multiple-comparison-corrected p > 0.25. Values shown in parenthesis indicate the value of p without correction for multiple comparisons. Neither Males nor Females show a pattern of PAF correlating with Accuracy in Precise Task-type trials. For Males, there is indication of a positive correlation between PAF and Accuracy in Relative trials. A significant difference between Male and Female correlations—indicated in bold—is present for the 3-Sample Relative Task-type during the Pre-Test Task Period (z = 2.56, p = 0.01).*

## Discussion

The present study presents evidence for sex-differences in the modulation of PAF during visuospatial working memory. Across four different Task-types, Females modulated PAF more than Males. In Males, PAF during task performance could be described as more constant. In Males, overall PAF across Baseline and working memory Maintenance periods was more highly correlated with task performance than it was in Females.

While PAF has been described as a “stable neurophysiological trait” in adults (Grandy et al., 2013), this phrase is not an accurate descriptor as it applies to adult females. In females, resting-state PAF varies predictably in relation to the menstrual cycle, as discussed in the Introduction. Additionally, the difference between resting-state and task-related PAF has also been found to vary across individuals and within individual females across the menstrual cycle (Bazanova et al., 2014). In the present study, we find that females, as a group, also modulate PAF between time periods during performance of a task.

What the cognitive function of this modulation may be, however, is not clear. Modulation did not render a behavioral advantage for females. Between females and males, there was no statistical difference in task performance – either for Accuracy or Response Time – and group performance distributions were quite similar. A recent meta-review that focused on sex differences in visuo-spatial working memory found small effects of a female advantage for specific location memory and a male advantage for other visuo-spatial tasks (Voyer et al., 2017). The present task, however, which contrasts working memory for precise and relative spatial locations, did not find a female or male advantage for either task. Another recent review (Grissom and Reyes, 2019) examined the evidence for sex and gender differences in executive function in both animal models and human behavior. They highlight the importance of considering motivation, reward, and strategy usage when evaluating whether small effects should reasonably be attributed to differences in sex or gender. In the present study, there are main effects of Task-type in both Accuracy and Response Time (Table 1), with lower Accuracy and slower Response Times, generally, in 3-Sample compared to 2-Sample Task-types. No speed-accuracy trade-off was present across Task-types, nor were there different patterns of behavior by sex group that might indicate differences in motivation or response to reward.

In Females, there was no significant relationship between either PAF Modulation and task performance, or overall PAF and task performance. For Males, though, indication of a relationship between PAF and task performance was present. Specifically, in males, Accuracy within Relative, but not Precise, Task-types was positively correlated with PAF.

As reviewed in the Introduction, a study with predominantly male participants found that the better performing half of participants had higher PAFs than the worse performing half (Klimesch et al., 1993). In a study comprised solely of males, a positive correlation was found between PAF and an aspect of “non-verbal intellect” as assessed by a standardized test (Bazanova and Aftanas, 2008). It would be prudent for future research, given the sex-specific findings here and the findings of prior studies, to account for sex as a factor when examining PAF and potential relationships with cognitive processes or task performance.

In the paradigm utilized here, Task-types varied—in working memory load demand and in the type of information required to be maintained. During both the Baseline and Maintenance time periods, only a fixation cross was visually present on screen. The amount of information a Participant needed to hold in mind, however, increased considerably in the Maintenance period compared with Baseline. Across Task-types, rules needed to be maintained, but with each trial, new stimuli-specific information needed to be maintained. There was no effect of Task-type on either PAF during Maintenance, or Modulation of PAF. However, the significant positive correlation in males between PAF, particularly during Baseline and performance on the higher-load Relative Task-type raises interesting questions.

The Baseline time period preceded the presentation of stimuli pertaining to the current trial and followed the inter-trial interval of the preceding trial. It was not a period of true “rest,” but rather a brief pause in which the cognitive demands of one trial have passed and the demands of a new trial are pending. How alert and attentive an individual participant was during this time period prior to the more demanding high-load Relative trials may have had an impact on trial performance. Some studies dividing alpha activity into upper and lower bands have associated a reduction in the lower range of alpha power with attentional processes – specifically alertness and expectancy (Klimesch et al., 1998; and see reviews: Klimesch, 1999; Bazanova and Vernon, 2014). That PAF falls at a higher frequency for some individuals might be explained by reduced lower alpha power due to increased alertness and expectancy.

The PAF provides only a single metric of the power distribution of alpha oscillatory activity and does not allow for conclusions to be drawn regarding the origin of variations in those distributions. We do not know, for example, whether PAF Modulation in females reflects a change in the rate of oscillations occurring within a single circuit or network, or whether it reflects a decrease in the activity of one circuit or network and an increase in the activity of another. A recent study utilizing MEG, examined changes in the frequency of localized oscillatory activity as they contributed to working memory performance (Noguchi and Kakigi, 2020). They found better working memory performers exhibited more “flexible modulation” of localized frequency based on memory load demands. Of the 24 participants they studied, 18 were female. The results of the current study indicate the peak frequency of alpha oscillatory activity across time periods changes more in females than in males, but a relationship between peak frequency and performance was only observed in males. Perhaps females were able to more rapidly modulate attention (and thus PAF) across the different time periods of each trial, whereas males had a more constant attention state across trials. It is important for future research to consider sex in order to understand whether the alpha frequency distribution or its modulation is differentially related to cognitive task performance according to sex.

Throughout we have used the term “sex” as a potential factor of influence on neural activity. In instances where there is indication that sex-hormones are an associated, and perhaps even causal factor in differences between females and males, the term sex-difference may be appropriate. Gender as a term, however, encompasses many of the developmental events and life experiences that arise, in part, from the juxtaposition of biological sex and identity. Gender undoubtedly impacts experience, thinking, strategy, and behavior – and potentially the neural activity underlying them. In the present study, we did not find differences in task performance between females and males that might point toward differences in strategy, motivation, or other factors in which gender has been shown to impact behavior. A limitation of the present study, however, is the smaller sample size of male participants compared to female. Echoing the recommendations espoused by experts in sex and gender research (e.g., McCarthy and Arnold, 2008; Jordan-Young and Rumiati, 2012; Rippon et al., 2014; Rubin et al., 2020), future studies that attempt to disentangle effects of sex from those of gender would greatly increase our broader understanding of individual differences. Studies able to incorporate larger participant groups, balanced in number, would be ideal.

We hypothesized that females and males have different cognitive or neural processes related to oscillatory brain activity by which behavioral outcomes are accomplished. The results of our analyses of peak alpha frequency, measured while participants performed multiple variants of a spatial working memory task, provides support for this hypothesis.

Researchers in cognitive neuroscience are more often coming to recognize the importance of studying oscillatory activity utilizing PAF-based frequency bands that are defined for each individual. It is critically important that researchers also be aware of the implications of the potential for sex differences in task-related modulations within those frequency bands. Only by accounting for factors of sex, when appropriate, can we hope to move toward a deeper understanding of the brain basis of behavior.

## Data Availability Statement

The raw data supporting the conclusions of this article will be made available by the authors, without undue reservation.

## Ethics Statement

The studies involving human participants were reviewed and approved by Institutional Review Boards of both Johns Hopkins University and the Johns Hopkins Medical Institutions. The patients/participants provided their written informed consent to participate in this study.

## Author Contributions

TG and SC conceived the research. KB and SC collected the data. TG and TH designed and carried out analyses. All authors reviewed the manuscript.

## Conflict of Interest

The authors declare that the research was conducted in the absence of any commercial or financial relationships that could be construed as a potential conflict of interest.
